# Prediction of the severity of patients with chronic coronary syndrome

**DOI:** 10.2478/abm-2024-0020

**Published:** 2024-09-20

**Authors:** Editorial Office of Asian Biomedicine

**Affiliations:** Faculty of Medicine, Chulalongkorn University, Bangkok 10330, Thailand

The goals in the care of patients with chronic coronary syndrome (CCS) are to secure the diagnosis, assess the extent of disease, relieve symptoms, and prevent future cardiac events such as acute coronary syndromes, revascularization, or death [1]. The presence of left main coronary artery disease (LMCAD) and the occurrence of triple-vessel diseases (TVDs) are significant indicators for the need of proper intervention [2].

The currently recommended first-line diagnostic tests for CCS include coronary computed tomographic angiography. Since the test may not be readily available in resource-poor settings, this recommendation is not mandatory [3]. Exercise stress testing and electrocardiography should be undertaken in all patients to document the presence and extent of coronary disease.

Magnetic resonance imaging would be required for some selected patients with possible ischemic heart disease to assess the conditions of coronary arteries, the status of left ventricular function, and the potential needs for revascularization [4].

Anginal symptoms can be prevented by either beta blockers, calcium channel blockers, or long-acting nitrates, which can be used singly or in combination, depending on the ability to immediately relieve the symptoms and side effects [1, 5]. In addition, other measures such as improving lifestyle factors are needed to reduce the chances of disease progression. Cognitive behavioral interventions can be beneficial to help individuals achieve a healthy lifestyle. Exercise-based cardiac rehabilitation may be effective in assisting patients for controlling risks. A multidisciplinary health care team may be assembled to help patients accomplish desirable outcomes. The members in the team may include cardiologists, general practitioners, nurses, dietician, physiotherapists, psychologists, pharmacists, etc [5]. Influenza vaccination can reduce the possibility of hospitalization in these patients. Periodic follow-up is crucial to assess the stability of patients’ conditions. New onset of heart failure or left ventricular systolic dysfunction (LVSD) may be an early sign of significant LMCAD, which is associated with the need for prompt interventions [6]. Influenza vaccination can reduce hospitalization for coronary syndrome in elderly patients with chronic obstructive pulmonary disease [7].

Nuchanat and Methavigul in this issue reported a predictive model for LMCAD or TVD in patients with CCS [8]. According to their prediction score, patients with CCS who developed new onset of heart failure or LVSD and suspected CAD, ST elevation in aVR, ST elevation in V1, and lateral ST depression were associated with increased risk of LMCAD/TVD and needed prompt appropriate intervention. The novel prediction score could predict LMCAD/TVD in those patients with acceptable sensitivity, specificity, PPV, and NPV. The generation of the score only requires the patients’ symptoms of LVSD and electrocardiography. This can be advantageous to design further strategies to save lives.

The prediction score should be further evaluated in larger samples to establish its benefit in various clinical settings to document the utility of the clinical score.
